# The “dot-in-circle” sign in musculoskeletal mycetoma on magnetic resonance imaging and ultrasonography

**DOI:** 10.1186/2193-1801-3-671

**Published:** 2014-11-13

**Authors:** Teeranan Laohawiriyakamol, Pramot Tanutit, Kanet Kanjanapradit, Keerati Hongsakul, Shigeru Ehara

**Affiliations:** Division of Diagnostic Radiology, Department of Radiology, Faculty of Medicine, Prince of Songkla University, 15 Karnjanavanit Road, Hat Yai, Songkhla, 90110 Thailand; Department of Pathology, Faculty of Medicine, Prince of Songkla University, 15 Karnjanavanit Road, Hat Yai, Songkhla, 90110 Thailand; Department of Radiology, Iwate Medical University School of Medicine, 19-1 Uchimaru, Morioka, 020-8505 Japan

**Keywords:** Dot-in-circle sign, Mycetoma, Magnetic resonance imaging, Ultrasonography

## Abstract

This study aimed to present the ‘dot-in-circle’ sign, which indicates the typical magnetic resonance imaging (MRI) and ultrasonographic (USG) findings for mycetoma involving soft tissue and bone. A total of 8 cases with histopathological proof of mycetoma affecting the musculoskeletal system, and that were examined via MRI and/or coexistent diagnostic ultrasonography between 2004 and 2013 in Songklanagarind Hospital were included in this study. The ‘dot-in-circle’ sign on the MRI and USG images of all the patients was reviewed by two radiologists. The analytic method was descriptive. All cases of musculoskeletal mycetoma revealed the ‘dot-in-circle’ sign on MRI, which was seen as multiple, small, round- to oval-shaped hyperintense lesions separated and surrounded by a low-signal intensity rim (circle), and a tiny, central, low-signal focus (dot). An USG study was available in four patients, and all USG findings demonstrated the ‘dot-in-circle’ sign as a central hyperechoic area (dot) surrounded by hypoechoic tissue (circle). In conclusion, the ‘dot-in-circle’ sign is a typical feature on MRI and USG findings for the diagnosis of musculoskeletal mycetoma.

## Background

Mycetomas are chronic infections of the skin, underlying soft-tissues, sometimes bones, and rarely viscera, and they are caused by both bacteria (actinomycetomas) and fungi (eumycetomas) (Hay 2008). The organisms are usually soil or plant saprophytes that are only incidental human pathogens. Mycetoma is more frequent in tropical and subtropical regions (Fahal and Hassan 1992). In the African continent, particularly Sudan, the prevalence is highest (Fahal et al. 2014). However, the first description of mycetoma of the foot was made in the city of Madura, India in 1846 - hence, the eponym “Madura foot” has been used (Lewall et al. 1985). As a result of the increasing numbers of migrant populations, cases are reported not only in endemic regions, but also in Western countries (Ahmed et al. 2003; White et al. 2014). The disease commonly involves parts of the body that are in direct contact with soil during daily activities, particularly the feet, lower legs or hands, and, occasionally, the head or back. The traumatic inoculation of the causative organism into the subcutaneous soft tissue by sharp objects such as thorns is a well-described theory (Fahal et al. 2014). The development of the disease is usually gradual and slow, but also progressive. The deep soft-tissue and bony structures are eventually invaded (Fahal et al. 1997). The clinical features of a painless subcutaneous mass, draining sinuses and exuding grains constitute the classic triad of mycetoma (Fahal et al. 1997; White et al. 2014). However, based on such clinical features, it is difficult to make an early diagnosis due to rare constitutional disturbances until the late stage, which is characterized by chronic sinus formation, discharging grains, extensive soft tissue and bone damage with deformity, and functional disability at the initial site of infection, often necessitating amputation (Abd El Bagi 2003). Furthermore, clinical examination is not always sensitive enough to detect the extension of the disease along the different soft tissue planes and bones, as small lesions with a few sinuses may have a deep, massive network of sinus tracts (Fahal et al. 2014; van de Sande et al. 2014). Therefore, mycetoma has been described as a sinister disease (Abd El Bagi et al. 1999). It has recently been included in the World Health Organization’s list of neglected diseases and deserves attention as it can be prevented (Fahal et al. 2014). Moreover, mycetoma is frequently misdiagnosed as a neoplasm, neuropathic foot, or a chronic bacterial or tuberculous infection. Biopsy and microbiological cultures can provide a definitive diagnosis; however, both may be difficult to achieve, especially with fastidious organisms (Sarris et al. 2003). Most culture methods are time consuming, commonly contaminated and require experience to accurately interpret their results (Fahal et al. 2014; van de Sande et al. 2014). The treatment of mycetoma is often difficult, and it consists of combined medical and surgical therapy. As a consequence of the inability to define the accurate extension of the lesion during surgery and the increasing resistance to drug treatment by fungal organisms, an incomplete cure and recurrence after treatment are common (Jain et al. 2012; Fahal et al. 2014). An untreated mycetoma infection with a complicated secondary bacterial infection and sepsis can be fatal. For that reason, an early and accurate diagnosis is very important to prevent morbidity and mortality, and have a better therapeutic outcome of the disease. Imaging can offer an early and non-invasive diagnosis of mycetoma. The ‘dot-in-circle’ sign, seen on magnetic resonance imaging (MRI) and ultrasonography (USG) has recently been reported as a characteristic and highly specific sign for mycetoma (Sarris et al. 2003; Kumar et al. 2007; Sen and Pillay 2011; Jain et al. 2012; Fahal et al. 2014; White et al. 2014). However, only a small number of cases describing the ‘dot-in-circle’ sign have been reported, and, mostly, in case reports. Therefore, the aim of this study was to present the ‘dot-in-circle’ sign, which indicates the typical MRI and USG findings for the diagnosis of mycetoma involving soft tissue and bone.

## Methods

Our Institutional Review Board (IRB) approved this retrospective study with a waiver for obtaining informed consent from the patients. A review was conducted of the medical records in the Hospital Information System (HIS) to evaluate patients that were diagnosed with soft-tissue mycetoma and had undergone magnetic resonance imaging(MRI) and/or coexistent diagnostic ultrasonography(USG) at the Department of Radiology, Songklanagarind Hospital, a tertiary-level hospital in Southern Thailand, during the 2004 – 2013 period. Eight cases were included in this study. MRI studies were available for all patients and USG ones for four patients. MRI was performed using a 3 T MR imaging scanner (Achieva X-series, Phillips, Eindhoven, Netherlands) and a 1.5 T MR imaging scanner (Magnetom vision, Siemens, Erlangen, Germany). The MR sequences were obtained using a phased-array surface coil and consisted of T1-weighted, T2-weighted, proton-density (PD) and fat-suppression (STIR) images. Intravenous gadolinium contrast medium was also administered and used in fat-suppression T1-weighted images. The USG obtained images in different planes with a diagnostic ultrasound system using linear scanners, 7.5 and 8 MHz transducers (Toshiba, Aplio500 ) as well as 9 and 12 MHz transducers (Philips, iU22). The images from MRI and USG studies were recorded in the Picture Archiving Communication System (PACS) of our institution. The studies were reviewed by two diagnostic radiologists with an experience of 5 and 10 years. The ‘dot-in-circle’ sign was reviewed on MRI as a sign of a small round- to oval-shaped T2-weighted hyperintense lesion with surrounding low-signal intensity and a central low-signal dot. It was also observed on the diagnostic ultrasound images as a central hyperechoic area surrounded by hypoechoic tissue. All cases underwent histopathological examination and were proven to be mycetoma. The demographic data and clinical histories of the patients were also collected. The analytic method was descriptive, using percentages and tables.

## Results

**Figure 1:**
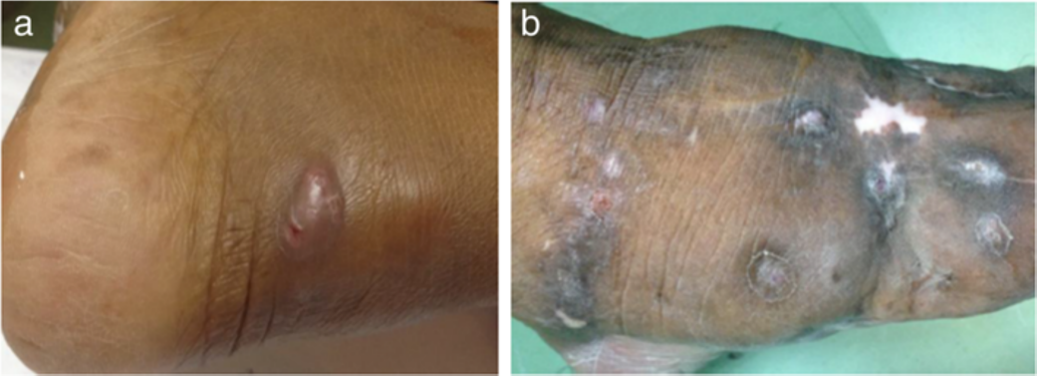
**Skin sinuses: (a) Skin sinuses with soft tissue swelling at the posterior aspect of the foot, (b) Skin sinuses with hyperpigmentation and encrustation at the dorsal aspect of the foot.**

**Figure 2:**
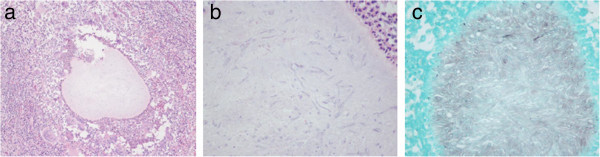
**Histological slides of eumycetoma: (a) Haematoxylin and eosin (H&E) stain, low magnification, shows collection of organisms in the subcutaneous tissue with surrounding infiltration of neutrophils and macrophages, (b) H&E stain, high magnification, shows cluster of septate hyphae form of fungal organisms, (c) GMS staining shows positive for fungal organisms.**

**Figure 3:**
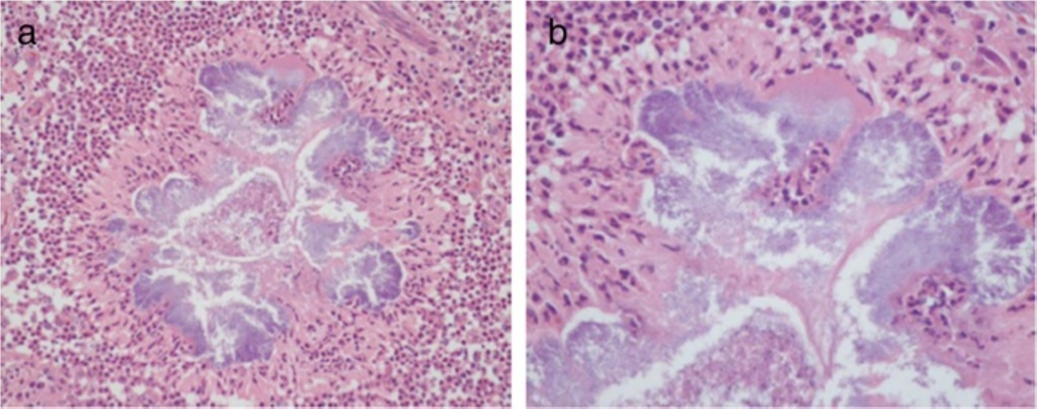
**Histological slides of actinomycetoma: (a) Haematoxylin and eosin (H&E) stain, low magnification, shows clumps of bacteria in the subcutaneous tissue with surrounding infiltration of neutrophils, (b) H&E stain, high magnification, shows clumps of filamentous bacteria.**

**Figure 4:**
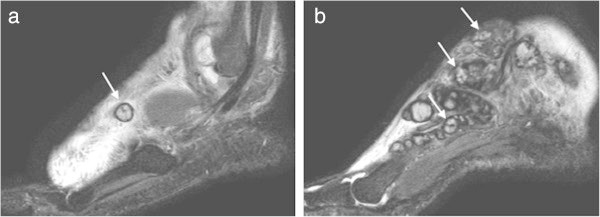
**MRI findings of the foot with mycetoma: (a) and (b) Sagittal STIR MR images show multiple, small, round-to-spherical hyperintense lesions separated by peripheral hypointense tissue.** Some of the lesions contain a central hypointense dot, resulting in the ‘dot-in-circle’ sign (arrows). The surrounding hyperintense inflammatory soft-tissue changes are also seen.

**Figure 5:**
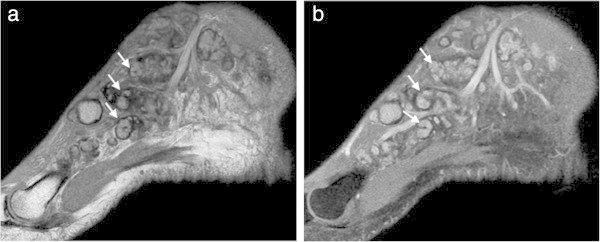
**MRI findings of the foot with mycetoma: (a) Sagittal T1-weighted MR image without contrast agent shows multiple isointense-to-hyperintense lesions containing some central hypointense dots and surrounded by hypointense tissue (arrows), (b) Sagittal T1-weighted MR image with fat suppression following intravenous gadolinium contrast administration shows enhancement of the lesions and persistent small, central, low-signal foci surrounded by tissue of low-signal intensity (arrows).**

**Figure 6:**
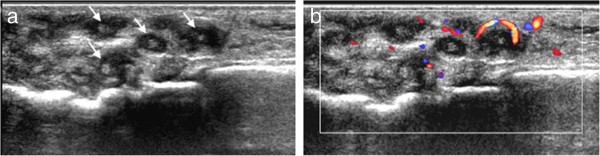
**Ultrasonographic images of the foot with eumycetoma: (a) the image shows multiple hypoechoic thick-walled lesions containing small, sharp, hyperechoic foci (arrows), (b) Color Doppler image shows increased vascularity of the surrounding inflammatory soft tissue.**

**Figure 7:**
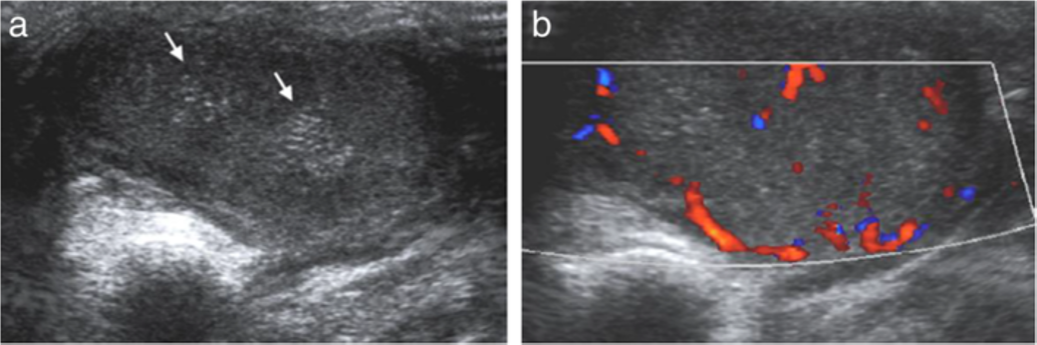
**Ultrasonographic images of the foot with actinomycetoma: (a) the image shows haziness and close aggregation of hyperechoic grains (arrows), (b) Color Doppler image shows prominent vascularity of the lesion.**

The general clinical data of the patients are summarized in Table 1. There were 8 patients in this study, consisting of 3 males and 5 females. The mean age of the patients was 42.25 years (27–60 years). Most patients were farmers or living in rural areas of the Southern Region of Thailand. All of them had a clinical history of soft-tissue swelling and complained of a mass at the involved location. Half of the patients reported no pain, and the same proportion had skin sinuses (Figure 1a-b). The duration of their clinical presentations ranged from 4 months to 17 years. No underlying disease was presented in all patients. Surgical treatment was repeated in 6 cases (75%) due to clinical recurrence and a lack of a definite diagnosis from microbiological examinations. The location of mycetoma was mainly in the foot (6 cases). All of the cases were histologically proven by biopsy. There were 4 cases with actinomycetoma and 4 cases diagnosed with eumycetoma. Histological analysis revealed central collection of the fungal element surrounded by inflammatory cells in both eumycetoma (Figure 2a-c) and actinomycetoma (Figure 3a-b). All patients underwent magnetic resonance (MR) examinations in order to characterize lesion and evaluate the extent of the disease. The ‘dot-in-circle’ sign was present in all 8 cases. The lesions were most clearly seen on the T2-weighted, STIR and proton-density sequences, as well as fat-suppressed T1-weighted sequence following intravenous gadolinium. These MR images showed conglomerate masses forming as a result of the merging of multiple small discrete round- to oval-shaped hyperintense lesions, measuring a few millimeters across each, involving the subcutaneous tissue, muscles, and intermuscular fascial planes at the affected location (Figure 4a-b and Figure 5a-b). The lesions were separated by a hypointense rim. Additionally, a central hypointense dot was observed within many of these lesions. Marrow abnormalities, representing osteomyelitis, were recognized in 2 cases, which, on MRI, appeared as an altered signal at the third metatarsal bone in one case as well as at the cuboid and navicular bones with an intraosseous ‘dot-in-circle’ sign in another case. The MRI findings were not significantly different in both patient’ groups (eumycetoma and actinomycetoma). Likewise, the ‘dot-in-circle’ sign was also observed on diagnostic ultrasonography (USG) imaging that was performed in four cases. In all of them, the USG images revealed a central hyper-reflective echoic area surrounded by hypo-reflective echoic tissue, and an increased vascularity on color Doppler images. However, different USG findings were observed between eumycetoma and actinomycetoma in all our four cases. In eumycetoma lesions, the grains appeared as numerous and very sharp hyperechogenicities with single or multiple, hypoechoic, thick-walled cavities (Figure 6a-b). In contrast, the hyperechoic grains were fine, hazy and closely-aggregated in actinomycetoma (Figure 7a-b).

**Table 1:** **General clinical data of patients**

Patient no.	Sex	Age (years)	Occupation	Clinical symptoms	Location	Histopathological diagnosis
1	Female	37	Government official	Nontender soft-tissue mass for 4 months	Right foot	Actinomycetoma
2	Female	28	Industrial employee	Nontender soft-tissue mass and skin sinuses for 1 year	Right arm	Actinomycetoma
3	Male	27	Private-sector employee	Recurrent tender soft-tissue mass for 1 year	Left leg	Eumycetoma
4	Male	46	Farmer	Tender soft-tissue swelling and skin sinuses for 2 years	Right foot	Actinomycetoma
5	Male	44	Farmer	Recurrent nontender soft-tissue mass and skin sinuses for 17 years	Right foot	Eumycetoma
6	Female	48	Farmer	Nontender soft-tissue mass for 1 year	Right foot	Actinomycetoma
7	Female	60	Farmer	Recurrent tender soft-tissue mass for 10 years	Left foot	Eumycetoma
8	Female	48	Farmer	Tender soft-tissue mass and skin sinuses for 6 months	Right foot	Eumycetoma

## Discussion

The clinical diagnosis of mycetoma is not often considered by physicians unless the pathognomonic clinical triad of painless subcutaneous nodules, fistulae, and purulent granular exudates developing several weeks after inoculation with the causative organism are noted (Magana 1984). However, in the presence of the typical clinical features of the discharge, the color of grains can be suggestive of the type of mycetoma. The grains of eumycetoma are usually black or pale, while those of actinomycetoma are white, yellow or red (Ahmed et al. 2004). According to literature, the disease prevalence is high in farmers and most frequently found in patients aged 20–40 years (Al-Heidous and Munk 2007; Parker et al. 2009; Fahal et al. 2014), as seen in our study. The male to female ratio is about 3.5-5:1 (Al-Heidous and Munk 2007; Fahal et al. 2014). The commonest site is the foot, representing 70% of cases (Foltz and Fallat 2004), but the hand, thigh, head and back have also been described (Cherian et al. 2009). The infection tends to occur after a trivial penetrating injury such as a thorn prick, and in most cases, such episodes are undetected (Shaw et al. 1994).

An initial diagnosis is often made after clinical assessment. However, the clinical entity is usually misdiagnosed as soft tissue tumors in the early stage as well as chronic bacterial or tuberculous infections when typical discharging sinuses are not noted (Cherian et al. 2009; Fahal et al. 2014). Furthermore, clinical examination alone is not suitable to assess the spread of the disease to deep soft tissue and bone. The frequent diagnosis at an advanced stage with significant destructive outcome makes us to realize the importance of an early diagnosis. Cultures are always necessary for a definitive diagnosis and an identification of the causal agents of mycetoma is considered to be the gold standard; however, some agents are difficult to identify by morphology alone (van de Sande et al. 2014). In addition, the culture technique is time consuming and often suffers from contamination (Shaw et al. 1994; Fahal et al. 1997). Currently, only molecular methods, i.e., polymerase chain reaction (PCR) and DNA sequencing, are able to identify the causative agent to the species level reliably (Ahmed et al. 2003; van de Sande et al. 2014). However, these methods are usually too expensive in endemic countries and often available in large medical centers (Fahal et al. 2014, van de Sande et al. 2014). Therefore, fast and affordable diagnostic tools are crucial for an early detection. Magnetic resonance imaging (MRI) and ultrasonography (USG) are helpful in the early diagnosis and help in discriminating mycetoma from other conditions (Fahal et al. 1997; Sarris et al. 2003; Kumar et al. 2007; Cherian et al. 2009; Parker et al. 2009; Jain et al. 2012; van de Sande et al. 2014; White et al. 2014).

The ‘dot-in-circle’ sign is clearly visible, characteristic and highly specific to diagnose mycetoma on both MRI and USG (Sarris et al. 2003; Kumar et al. 2007; Cherian et al. 2009; Parker et al. 2009; Sen and Pillay 2011; Jain et al. 2012; Fahal et al. 2014). This sign was first suggested by Sarris et al. (2003) in two cases of soft tissue mycetoma of the foot on MRI. They observed multiple small lesions of high signal intensity separated by a low-intensity stroma (circle), and a small low-intensity focus (dot) in the center of these lesions on T2-weighted, STIR and T1-weighted, fat-saturated, gadolinium-enhanced images. Other reports as well as our study (Kumar et al. 2007; Cherian et al. 2009; Parker et al. 2009; Sen and Pillay 2011; Jain et al. 2012; Fahal et al. 2014; White et al. 2014) have shown similar MRI findings of the ‘dot-in-circle’ sign in all of the investigated cases. The MRI characteristics of musculoskeletal mycetoma demonstrate the ‘dot-in-circle’ sign, particularly on fluid-sensitive sequences, STIR and proton-density sequences, and T1-weighted fat-saturated images with gadolinium enhancement. Nevertheless, the usefulness of MRI with gadolinium enhancement in routine soft tissue infections remains debatable. Sharif et al. (1991) have reported that gadolinium enhancement is of limited value.

Histologically, the eight cases in our study, similar to a previous report (McElroy et al. 1992) revealed the typical features of mycetoma, comprising a granulomatous area with a purulent center containing characteristic grains, surrounded by a thick, fibrous capsule. The findings of a granulomatous inflammatory reaction to mycetoma are non-specific; however, the grain is useful for the diagnosis and identification of causative agents (Czechowski et al. 2001). Sarris et al. (2003) and Cherian et al. (2009), who discussed the correlation of MRI and reviewed histological findings, suggested that the high-signal foci seen on MRI represent the inflammatory granulomata, the low-signal tissue seen surrounding these lesions represent the fibrous matrix, and the small central hypointense foci within the granulomata represent the fungal balls or grains. The initial report by Czechowski et al. (2001) observed the MRI findings of mycetoma as small lesions of low-signal intensity on T1- and T2-weighted images, and assumed that these appearances were due to susceptibility from the metabolic products of the “grains”.

Even though MRI finding is non-specific (Seeger et al. 1991; Locken et al. 1997), the ‘dot-in-circle’ sign in the MRI is known to be pathognomonic of mycetoma, and it can be used to detect the lesion extent, as well as to help in planning appropriate treatment strategies. El Shamy et al. (2012) recently reported a new MRI grading system for the diagnosis and management of mycetoma. Mycetoma lesions can be classified as mild, moderate or severe based on scores for skin and subcutaneous tissue, muscle and bone involvements. Nevertheless, the differentiation between the eumycetoma and the actinomycetoma in soft-tissue infection is not possible on MRI (Kumar et al. 2007; Sen and Pillay 2011; El Shamy et al. 2012). Although eumycetoma frequently reveals soft-tissue macroabscesses with bone cavitation, and actinomycetoma more often shows soft-tissue microabscesses, periosteal reaction and reactive sclerosis, such differences are not statistically significant (El Shamy et al. 2012). In addition, the soft-tissue hemangiomas may have the ‘dot-in-circle’ sign on MR imaging, and that differentiation may be occasionally difficult without a clinical history. The reported case by Petscavage et al. (2010) was misdiagnosed as a soft-tissue hemangioma on MRI due to the presence of serpiginous rather than round enhancing masses with the ‘dot-in-circle’ sign (the “dots” were mistaken for phleboliths). Another mimic of ‘dots’ are rice bodies, noted as small low-signal intensity foci in the synovial fluid of patients with articular or tendinous tuberculosis and rheumatoid arthritis (Jaovisidha et al. 1996; Hsu et al. 2004). There is also a limitation of the MRI method in the standard dignosis of mycetoma because it is not readily available. If the technique is available, expertise is required to differentiate between mycetoma and other soft-tissue tumors, chronic bacterial and tuberculous infections (van de Sande et al. 2014).

Another specific imaging technique used to detect mycetoma is the USG (Fahal et al. 1997, 2014; Sen and Pillay 2011; van de Sande et al. 2014). This examination is useful in an early diagnosis and a pre-operative planning. In addition, it is simple, safe, fast and reproducible, and it is routinely used in the diagnosis of mycetoma. Because mycetoma lesions have characteristic USG appearances, it can differentiate mycetoma, including eumycetoma and actinomycetoma from other conditions (Fahal et al. 1997, 2014). Fahal et al. (1997) have demonstrated *in vitro* imaging of mycetoma lesions where the hyper-reflective echoes correspond to grains, as well as the USG differentiation between eumycetoma and actinomycetoma. In eumycetoma lesions, the grains produce numerous, sharp, hyper-reflective echoes, consistent with black grains and a grain cement substance, and there are single or multiple thick-walled cavities with no acoustic enhancement. In actinomycetoma, the findings are similar, but the hyper-reflective echoes are fine, closely-aggregated and commonly settle at the bottom of the cavities. The less distinct hyper-reflective echo of actinomycetoma grains may be due to their consistency, smaller size, individual embedding of the grains and the absence of a cement substance in some types of actinomycoma. The USG appearances seen in four cases of our study also revealed the ‘dot-in-circle’ sign as a central hyperechoic area (dot) surrounded by hypoechoic tissue (circle), with differences on USG images between eumycetoma and actinomycetoma as reported previously (Fahal et al. 1997, 2014; Sarris et al. 2003; Parker et al. 2009). Eventually, the ultrasonographic ‘dot-in-circle’ sign is comparable to the MRI ‘dot-in-circle’ sign (Fahal et al. 1997; Cherian et al. 2009; Sen and Pillay 2011; van de Sande et al. 2014), with multiple, round, hypoechoic inflammatory granulomatous lesions containing hyperechoic foci of mycetoma grains.

## Conclusion

In conclusion, this study suggests that both MRI and USG can aid in the early diagnosis of mycetoma when they demonstrate the imaging features of the ‘dot-in-circle’ sign corresponding to inflammatory granuloma with a central fungal grain. In line with the limitation of previous studies with a small number of cases describing the ‘dot-in-circle’ sign, and with the increasing use of MRI and widespread availability of USG to evaluate soft-tissue lesions, our study contributes to the data on the specificity of the “dot-in-circle” sign, which indicates the typical MRI and USG findings for the diagnosis of mycetoma involving soft tissue and bone. In addition, we believe this sign is an important imaging clue to distinguish mycetoma from other lesions.

## Abbreviations

MRI: Magnetic resonance imaging
USG: Ultrasonography
PD: Proton-density
STIR: Short tau inversion recovery
PACS: Picture archiving communication system
PCR: Polymerase chain reaction.

## References

[CR1] Abd El Bagi ME (2003). New radiographic classification of bone involvement in pedal mycetoma. Am J Roentgenol.

[CR2] Abd El Bagi ME, Sammak BM, Al Shahed MS, Yousef BA, Demuren OA, Al Jared M, Thagafi MA (1999). Rare bone infections “excluding the spine”. Eur Radiol.

[CR3] Ahmed AO, Desplaces N, Leonard P, Goldstein F, Hoog SD, Verbrugh H, van Belkum A (2003). Molecular detection and identification of agents of eumycetoma: detailed report of two cases. J Clin Microbiol.

[CR4] Ahmed AO, van Leeuwen W, Fahal A, van de Sande W, Verbrugh H, van Belkum A (2004). Mycetoma caused by *Madurella mycetomatis*: a neglected infectious burden. Lancet Infect Dis.

[CR5] Al-Heidous M, Munk PL (2007). Radiology for the surgeon: musculoskeletal case 40. Can J Surg.

[CR6] Cherian RS, Betty M, Manipadam MT, Cherian VM, Poonnoose PM, Oommen AT, Cherian RA (2009). The “dot-in-circle” sign-a characteristic MRI finding in mycetoma foot: a report of three cases. Br J Radiol.

[CR7] Czechowski J, Nork M, Haas D, Lestringant G, Ekelund L (2001). MR and other imaging methods in the investigation of mycetomas. Acta Radiol.

[CR8] El Shamy ME, Fahal AH, Shakir MY, Homeida MM (2012). New MRI grading system for the diagnosis and management of mycetoma. Trans R Soc Trop Med Hyg.

[CR9] Fahal AH, Hassan MA (1992). Mycetoma. Br J Surg.

[CR10] Fahal AH, Skeik HE, Homeida MMA, Arabi YE, Mahgoub ES (1997). Ultrasonographic imaging of mycetoma. Br J Surg.

[CR11] Fahal AH, Shaheen S, Jones DHA (2014). General orthopaedics: the orthopaedic aspects of mycetoma. Bone Joint J.

[CR12] Foltz KD, Fallat LM (2004). Madura foot: atypical finding and case presentation. J Foot Ankle Surg.

[CR13] Hay RJ, Wolff K, Goldsmith LA, Katz SI, Gilchrest BA, Paller AS, Leffell DJ (2008). Deep fungal infections. Fitzpatrick’s Dermatology in General Medicine.

[CR14] Hsu CY, Lu HC, Shih TT (2004). Tuberculous infection of the wrist: MRI features. Am J Roentgenol.

[CR15] Jain V, Makwana GE, Bahri N, Mathur MK (2012). The “dot-in-circle” sign on MRI in maduromycosis: a characteristic finding. J Clin Imaging Sci.

[CR16] Jaovisidha S, Chen C, Ryu KN, Siriwongpairat P, Pekanan P, Sartoris DJ, Resnick D (1996). Tuberculous tenosynovitis and bursitis: imaging findings in 21 cases. Radiology.

[CR17] Kumar J, Kumar A, Sethy P, Gupta S (2007). The dot-in-circle sign of mycetoma on MRI. Diagn Interv Radiol.

[CR18] Lewall DB, Ofole S, Bendl B (1985). Mycetoma. Skeletal Radiol.

[CR19] Locken JA, Strong B, Martin TP (1997). Mycetoma of the calf. Skeletal Radiol.

[CR20] Magana M (1984). Mycetoma. Int J Dermatol.

[CR21] McElroy JA, de Almeida C, Su WP (1992). Mycetoma: infection with tumefaction, draining sinuses and grains. Cutis.

[CR22] Parker L, Singh D, Biz C (2009). The dot-in-circle sign in Madura foot. J Foot Ankle Surg.

[CR23] Petscavage J, Richardson ML, Jonelle M (2010). Madura foot masquerading as a hemangioma. Radiol Case Rep.

[CR24] Sarris I, Berendt AR, Athanasous N, Ostlere SJ (2003). MRI of mycetoma of the foot: two cases demonstrating the dot-in-circle sign. Skeletal Radiol.

[CR25] Seeger LL, Dungan DH, Eckardt JJ, Bassett LW, Gold RH (1991). Nonspecific findings on MR imaging: the importance of correlative studies and clinical information. Clin Orthop.

[CR26] Sen A, Pillay RS (2011). Case report: Dot-in-circle sign - an MRI and USG sign for “Madura foot”. Indian J Radiol Imaging.

[CR27] Sharif HS, Clark DC, Aabed MY, Aideyan OA, Mattsson TA, Haddad MC, Ohman SO, Joshi RK, Hasan HA, Haleem A (1991). Mycetoma: comparison of MR imaging with CT. Radiology.

[CR28] Shaw CJ, Thomason AS, Spencer JD (1994). Fungal osteomyelitis of the foot: a report of an unusual case. J Bone Joint Surg.

[CR29] van de Sande WWJ, Fahal AH, Goodfellow M, Mangoub ES, Welsh O, Zijlstra EE (2014). Merits and pitfalls of currently used diagnostic tools in mycetoma. PLoS Negl Trop Dis.

[CR30] White EA, Patel DB, Forrester DM, Gottsegen CJ, O’Rourke E, Holtom P, Charlton T, Matcuk GR (2014). Madura foot: two case reports, review of the literature, and new developments with clinical correlation. Skeletal Radiol.

